# An Interpretable Predictive Model of Vaccine Utilization for Tanzania

**DOI:** 10.3389/frai.2020.559617

**Published:** 2020-10-30

**Authors:** Ramkumar Hariharan, Johnna Sundberg, Giacomo Gallino, Ashley Schmidt, Drew Arenth, Suvrit Sra, Benjamin Fels

**Affiliations:** ^1^ Macro-Eyes, Inc, Seattle, WA, United States; ^2^ College of Engineering, Northeastern University, Seattle, WA, United States; ^3^ Laboratory for Information and Decision Systems, Department of Electrical Engineering and Computer Science, MIT, MA, United States

**Keywords:** machine learning, forecasting, artificial intelligence, random forests, vaccine

## Abstract

Providing accurate utilization forecasts is key to maintaining optimal vaccine stocks in any health facility. Current approaches to vaccine utilization forecasting are based on often outdated population census data, and rely on weak, low-dimensional demand forecasting models. Further, these models provide very little insights into factors that influence vaccine utilization. Here, we built a state-of-the-art, machine learning model using novel, temporally and regionally relevant vaccine utilization data. This highly multidimensional machine learning approach accurately predicted bi-weekly vaccine utilization at the individual health facility level. Specifically, we achieved a forecasting fraction error of less than two for about 45% of regional health facilities in both the Tanzania regions analyzed. Our “random forest regressor” had an average forecasting fraction error that was almost 18 times less compared to the existing system. Importantly, using our model, we gleaned several key insights into factors underlying utilization forecasts. This work serves as an important starting point to reimagining predictive health systems in the developing world by leveraging the power of Artificial Intelligence and big data.

## Introduction

Vaccines have been touted as the “single most life-saving healthcare innovation ever” (Orenstein and Ahmed, 2017). It has also been emphasized that vaccination and not vaccines save lives (Breiman and Friedman, 1984). Additionally, a recent study on 94 low- and middle-income countries estimated that a $34 billion investment in immunization programs resulted in savings of $1.53 trillion in broad illness-related economic benefits (Ozawa et al., 2016). Maximizing immunization coverage for any population is an important public health goal for all countries and 194 Member States of the World Health Assembly in May 2012 agree, having developed a framework to prevent millions of deaths by 2020 through more equitable access to existing vaccines for people in all communities (WHO, Global Vaccine Action Plan 2012–2020).

One of the challenges that countries need to overcome to move closer to this goal is accurate forecasting of vaccine utilization (Meuller et al., 2016). Under-estimation of vaccine demand can lead to reduced coverage and vaccine stock-outs while over estimation leads to vaccine wastage (Meuller et al., 2016). The majority of existing vaccine utilization forecasting systems fall into one of two broad categories: 1) Routine data collection such as data on immunization and/or stock level changes entered by health workers (Logistimo, 2011) and past trends detected from immunization and/or stock level change data extrapolated to forecast future utilization; and 2) Population level data using population level survey data on pregnant women and child births (John Snow, Inc. and USAID, 2010) extrapolated based on an age-based vaccination scheme and then used to calculate utilization. A recent study had also used a discrete event simulation model to predict the effect of introducing a demand forecasting system into a low-income country’s supply chain (Mueller et al., 2016).

However, as pointed out by multiple studies, existing vaccine utilization forecasting systems are far from perfect and have large room for improvement (Patel et al., 2015; Lydon et al., 2017, Path and World Health Organization report, 2011). These significant inaccuracies in forecasting vaccine utilization may stem from data inaccuracies. For example, the error inherent in attempting to extrapolate population census data which is often vastly outdated. Also, leading to inaccuracies in forecasting vaccine utilization is the use of univariate forecasting models that do not take into account the multivariate nature of the problem inherent in its scope. For example, vaccine utilization at any given health facility is driven by factors apart from the catchment population. Factors may include the characteristics of the facility such as ease of access as reflected in its geo-coordinates, type of facility such as private or public, altitude, etc. These two challenges can be addressed by 1) use of actual vaccine utilization data for each health clinic as this data is very close to ground reality and is recent; and 2) building and applying a multivariate machine learning approach that not only uses vaccine utilization data, but also leverages other kinds of data about the health facility and population to predict vaccine utilization.

Here, we used recent vaccine utilization data, together with publicly available highly multivariate health facility data to forecast individual health facility level vaccine utilization in two Tanzania regions. Importantly, to accurately forecast vaccine utilization, we trained and applied a powerful machine learning model: a Random Forest Regressor (RFR). Our approach not only gives accurate vaccine utilization forecasts, but also provides insights into the data itself. Our findings have clinical and global health program relevance because accurate forecasting of utilization down to facility level will serve to reduce vaccine wastage and stock-outs, in turn contributing to optimal vaccine deployment and the most efficient use of resources.

To the best of our knowledge, this paper is the first to leverage regionally and temporally relevant utilization data together with a host of other features to forecast vaccine utilization.

We worked with the Ministry of Health of Tanzania and an NGO partner, PATH, with experience in the region and specifically vaccines to pilot our approach in three regions of Tanzania.

## Materials and Methods

We used Python v3.6 (Python Software Foundation, 2018), and Jupyter notebook v5.4.0 (Perez and Granger, 2007) for all calculations and plots. Specifically, for data pre-processing, we used the fastai library (v1.0, Howard and Gugger, 2020), whereas for machine learning and model performance evaluation, we relied on scikit-learn v0.21 (Pedregosa et al., 2011). We used seaborn and matplotlib for generating plots of data (Seaborn, Matplotlib).

### Data Gathering and Feature Augmentation

We used a diverse set of features or variables to forecast vaccine utilization. We obtained daily vaccine utilization data from 710 health facilities across three different regions in Tanzania—Arusha, Tanga and Kilimanjaro. The data had 13 features for every observation. We split the datetime feature into 12 features using the fastai add-datepart function (Howard and Gugger, 2020). While these features can partially contribute to predicting vaccine consumption, we believed that analyzing additional features describing individual facilities and their catchment populations would improve predictive performance. Therefore, we examined the Tanzania Health Facility Registry (THFR, see website reference) and the Tanzania National Bureau of Statistics (TNBS, see website reference) to extract several additional dimensions of data to augment our feature set. We also used a web-based tool (GPS visualizer, see website reference) to add elevation to each health facility. The data we used for building predictive models included several new features such as geo-coordinates, distance to nearest facility, type of facility and regional population (See Table 1 for a comprehensive list of features). Additionally, since recent vaccine utilization can serve as a useful feature, we used a rolling 3-month average as a feature. This resulted in a total of 32 features which went into our machine learning model. These features encompassed key intuitive vaccine utilization determinants — 1) Details of the nature of the vaccine, 2) details of each health facility and 3) features related to the catchment population around each facility.

**TABLE 1:** List of features used for predicting vaccine utilization in three regions of Tanzania.

Sl No	Name of feature
1	Facility ID^1^
3	Region^1^
4	District^1^
5	Ward^1^
6	Village/street^1^
7	Transaction description^1^
8	Vaccine type^1^
9	Change in stock^1^
10	Reason for change^1^
11	Immunization type^1^
12	Expiry^1^
13	Vaccine manufacturer^1^
14–25	12 features derived from immunization date (“year”, “month”, “week”, “day”, “dayofweek”, “dayofyear”, “Is_month_end”, “Is_month_start” “Is_quarter_end”, “Is_quarter_start”, “Is_year_end”, “Is_year_start”)*
26	Geo-coordinate I: Latitude^2^
27	Geo-coordinate II: Longitude^2^
28	Geo-coordinate III: Elevation^2^
29	Total regional population^2^
30	Type of facility^2^
31	Ownership^2^
32	Average utilization from a three-month rolling average calculation

**Table 1** is a comprehensive list of all 32 features we used for building the predictive model. Facility refers to a health facility. 1 indicates the data for the feature was obtained through PATH and Tanzania MoH. 2 indicates the data for the feature was obtained from other sources. Here, “*” indicates the vaccination date was split into 10 columns using the fastai’s add_datepart function (Tanzania Health Ministry Registry and Tanzania National Bureau of Statistics).

### Data Preprocessing

We used fastai modules—train_cats, add_datepart, and proc_df for initial data pre-processing (Howard and Gugger, 2020). Specifically, we carried out the following steps — 1) Assessing fraction null values for each column. No column had more than 4% null values, 2) For categorical variables, null values were treated as a separate level, and imputed cells were recorded in a separate variable, 3) Median values were used to impute missing continuous variables, 4) Date field was split into 12 separate fields such as time elapsed from the start of the year, is date start of month, etc. All date fields are documented elsewhere (fast.ai, see Howard and Gugger, 2020), and 5) Categorical mapping rules and imputation value to feature mappings were stored in a dictionary which was re-used for pre-processing test data.

### Data Ordering and Partitioning

We aggregated data per facility and vaccine type into biweekly utilization, which we attempted to forecast. The decision to forecast biweekly utilization was based on discussions with healthcare providers in Tanzania (internal communication). Additionally, summarizing the data into monthly or bimonthly rows does not give us sufficient data size to make robustly extrapolatable forecasts. A factor that led to this decision was the frequency of power outages in those regions. We trained, tuned, and evaluated an RFR to forecast biweekly vaccine utilization at a given health facility in Arusha, Tanga, and Kilimanjaro.

Further, approximately 70% of the data was used to train the model. We equally partitioned the remaining data into validation and test sets. The validation data was used for hyper-parameter tuning whereas the test set was used to report final performance scores. Since the goal was to predict future vaccine utilization, train-validation-test split was done in a temporally sorted manner making sure that the hold-out sets contained only data from dates that were in the future relative to the training, or the validation data.

### Measures of Model Performance and Optimization Function

We used two different measures to evaluate model performance, 1) Root Mean Square Error (RMSE) between predicted and actual biweekly utilization values, and 2) Fraction error (F. E), to measure how far each predicted target variable value was from the actual value of that variable. We also used F. E as the optimization function for our model. We define F. E as

F. E=|(A.U−P.U)|÷A.U

Where AU is actual biweekly utilization and P.U is predicted bimonthly utilization.

## Results

### Model Selection

We implemented a wide range of commonly used machine learning algorithms to select the best model. Among all the algorithms—regularized linear regression, support vector machine, k-nearest neighbors, RFR and autoregression based univariate time series models, random forests had the best performance on the validation set (Table 2). Our exploratory model building also included multilayered neural networks, including recurrent neural nets; however, the models 1) failed to yield comparable performances to some of the other classic machine learning models and, 2) gave largely uninterpretable predictions with no simple way to find feature importances. This bolstered our choice of RFR for vaccine utilization forecasting.

**TABLE 2:** Performances of different machine learning (ML) algorithms on our dataset.

Sl no	ML algorithm	RMSE
1	Random forest	17.0
2	Gradient boosting	17.2
3	K- nearest neighbors	19.1
4	Elasticnet	19.3
5	Support vector regression	22.4
6	ARIMA	24.7
7	Neural net (3 layered)	26.5

**Table 2** Performance, based on RMSE, of different hyper-parameter tuned machine learning models on the validation set.

### Using an Random Forest Regressor to Forecast Bi-Weekly Vaccine Utilization in Tanzania

We trained, tuned, and evaluated an RFR to forecast biweekly vaccine utilization at health facilities in Arusha, and Tanga. We chose RFR for further downstream analyses because, 1) As measured by the Root Mean Square Error (RMSE) RFR outperformed all five of the most commonly used machine learning algorithms and, 2) RFR is a powerful and generalizable predictive framework which can also be leveraged to understand the data better.

We used the validation data to set the values for 1) number of estimators, 2) max features, and 3) min samples leaf. Further, we optimized the model to obtain a minimal validation set RMSE. Our optimized values for these hyperparameters were 40, 0.95, and 7, respectively. We also set n_jobs = −1 in scikit-learn, to effectively utilize all available compute cores.

We made two different versions of our RFR (Leo Brieman, 2001) ↓ model I which uses all 32 features and model II which uses all 32 features except the 3-months rolling average. The two models are both based on random forests but differ in the number of features. Importantly, recent vaccine utilization averages are not used for forecasting in model II.

### Overall Performance of Our Models at Various Levels of F. E Threshold

**FIGURE 1:**
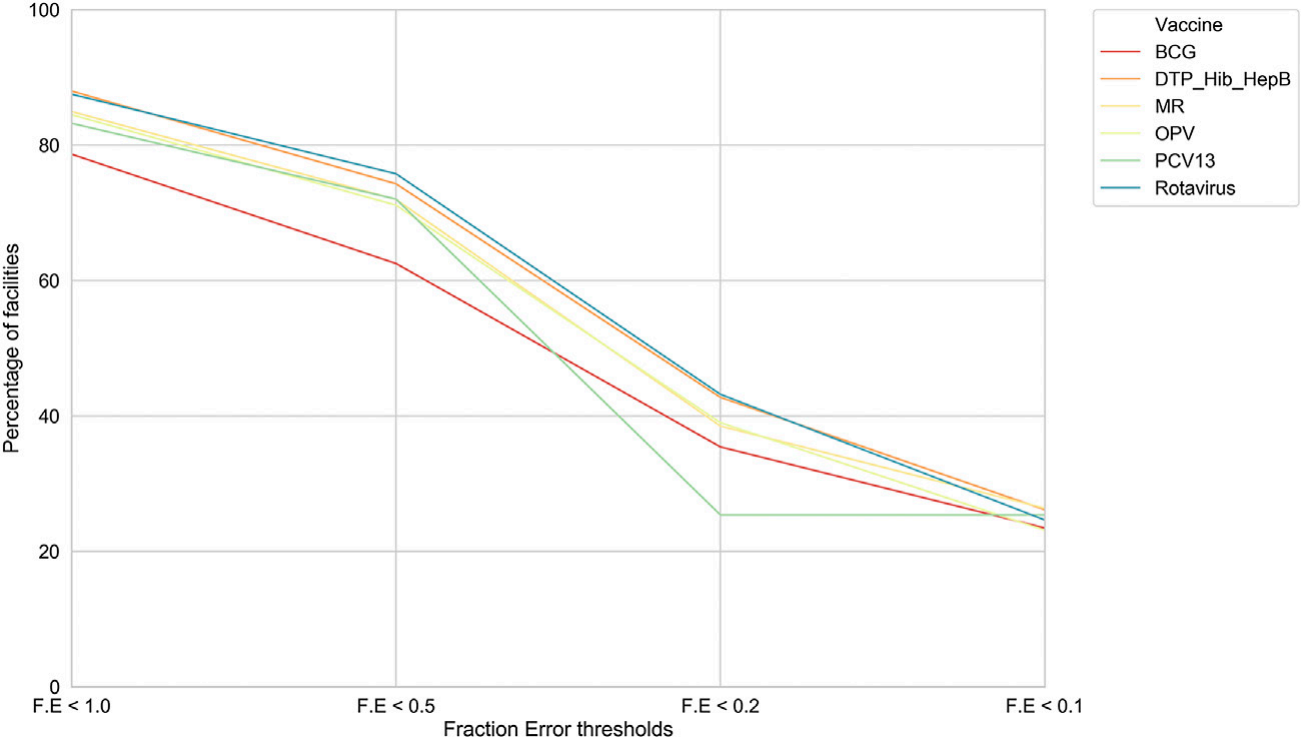
For model I, the percentage of health facilities in Tanzania meeting F. E thresholds. For each of the six vaccines, our model performances across facilities at different F. E thresholds.

Model I. We picked this optimized RFR and achieved an F. E of less than 0.2 for a significant subset of facilities, that is, an error of ±2 doses where actual vaccine utilization was 10 doses (Figure 1).

**FIGURE 2:**
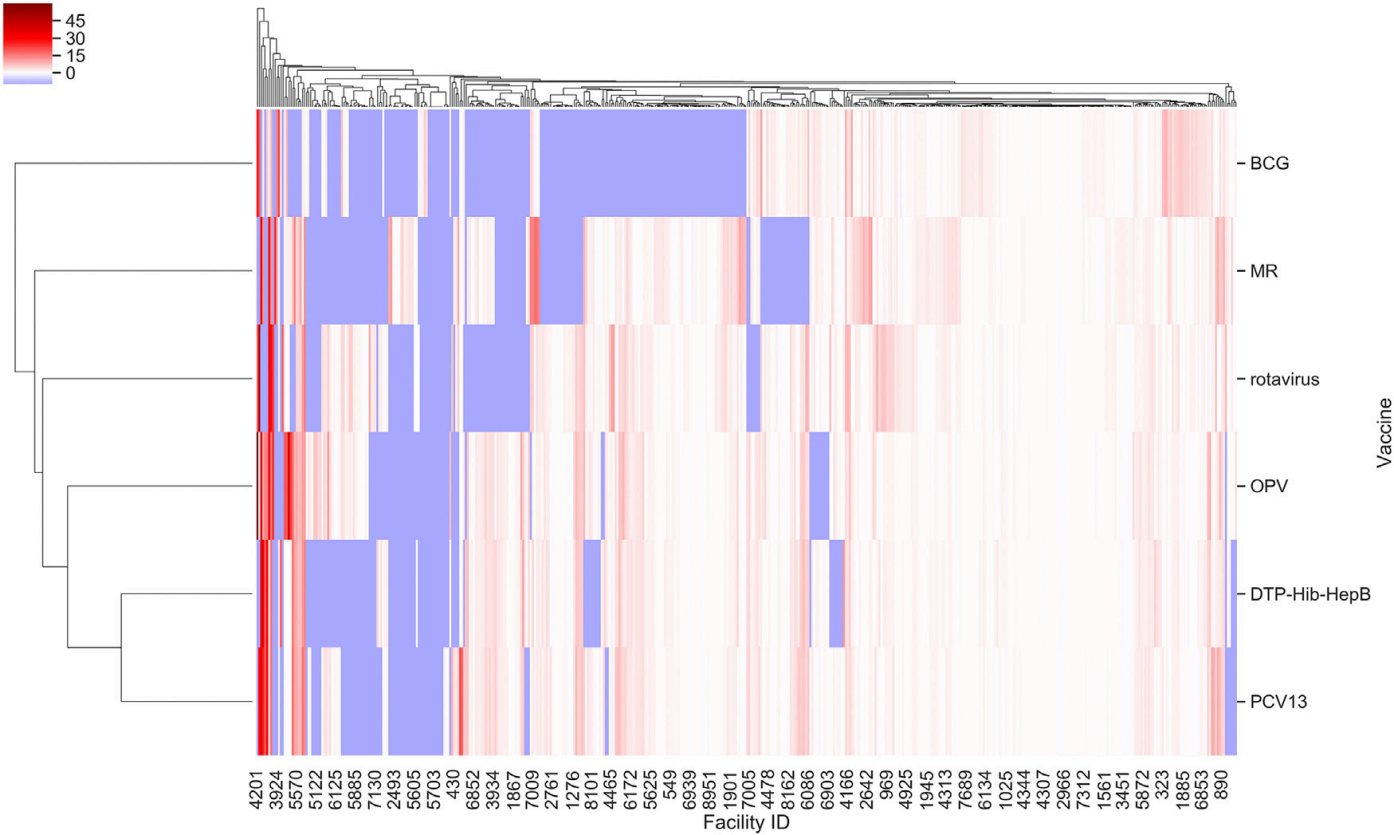
F. E for vaccine types across facilities in Tanzania based on model I. Values were derived from prediction of bi-weekly vaccine doses applying our random forest regressor. Facility ID annotations on the *x*-axis are sparse owing to space limitations.

Further, we found that for the majority of health facilities where our predictive performance passed a given threshold for one vaccine, it also passed the same threshold for the other vaccine as well. We were thus able to predict bi-weekly vaccine utilization within an F. E < 0.1 for 22–27% of the facilities depending on the vaccine (Figure 2). Our model performed best for rotavirus vaccine utilization, with almost 50% of facilities approaching a F. E < 0.2.

Model II. This version of the model is more generalizable since all its features can be derived from publicly available resources. It has an F. E < 0.2 for 15–20% of facilities (Supplementary Figure S1). Vaccine type was identified as the most important feature for this model (Supplementary Figure S2).

### Feature Importance Using Random Forest Regressor

We used the “mean decrease accuracy” method to calculate importance scores for all features in our model. This involved randomly permuting each column of data, and then calculating the decrease in *R* ^2^ on the out of the box datasets (Brieman and Friedman. 1984). This feature importance scoring scheme, as implemented in scikit-learn, outputs relative feature importance scores.

**FIGURE 3:**
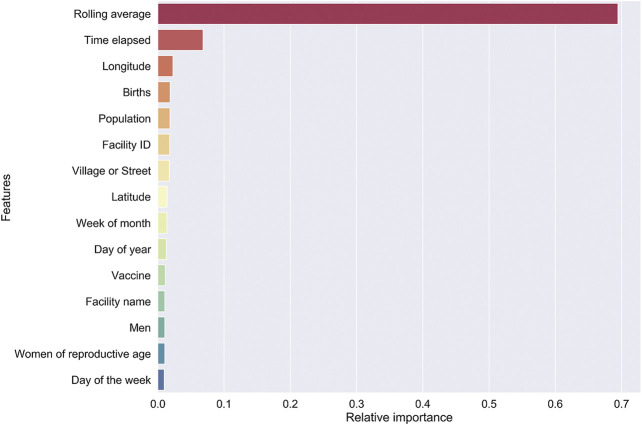
Feature importance for our RF regressor underlying model I was computed by “jumbling up” the data within each feature (= column) and calculating the change in *R* ^2^.

Model I. By far, the three-months utilization rolling average has the greatest impact on the model prediction. This is followed by the time, relative to year beginning, to vaccination. A number of features that relate to the health facility come next in our model—public or private, GIS coordinates, district, and ward. We hypothesize that features related to the facility implicitly encode demand characteristics of the catchment population (Figure 3).

Model II. Vaccine type was identified as the most important feature for this model (Supplementary Figure S3). Again, a number of features that relate to the health facility showed up next.

We made sure our model performance evaluation included completely non-overlapping “future” data. We included actual geo-coordinates and altitude to remove subjective levels like “High altitude” or “Low altitude”. Removing the geo-coordinates still gives us a meaningful model with only a small (0.7) decrease in forecasting accuracy. This is hardly surprising since the relative feature importance based on the Random Forest Regressor falls off rather sharply after feature #3 “Births”.

We hypothesize that features related to the facility implicitly encode demand characteristics of the catchment population.

### Comparisons With Existing Model

In order to get a measure of vaccine utilization forecasts based on the existing system, we mined stock addition data. We did not find evidence of any statistical forecasting model. The amount of new stock additions immediately prior to our validation and test dates, were treated as “forecasts” made by the baseline, existing system. Since we had actual utilization data for the validation and test period, we were able to calculate F. E and RMSE for the existing system. Evaluation of RFR model performance against this baseline was made on the basis of F. E and RMSE (see Table 3).

**TABLE 3:** Comparison of predictive performance and benchmarking.

Sl no	Predictive model or system	Avg RMSE	Avg F. E
1	Existing system	351	43.02
2	RFR model I	17.90	1.56
3	RFR model II	19.00	2.42

**Table 3** summarizes benchmarking of the two RFR models against the existing system. RFR model 1 includes 3 months moving average as a feature while model 2 does not.

The Global Alliance for Vaccines and Immunization anticipates a six-fold increase from 2010 to 2020 in the number of vaccine doses given to complete immunization. Global coverage for basic childhood vaccines has reached a record 86%, but there has been a parallel increase in vaccine wastage, decreasing resource efficiency. Vaccine stock-outs compound the problem by wasting opportunities for immunization. There is no good reason why the correlation between higher rates of immunization and supply chain waste should persist. Further, wastage of all kinds is largely a result of inaccurate, univariate or static models of vaccine demand assumptions; in summation the wrong quantity and type of vaccines at the wrong time. There is thus an enormous economic incentive to reduce vaccine wastage and stock-outs, without sacrificing high immunization coverage rates.

Random Forests (Leo Breiman, 2001) is an algorithm that uses bootstrap to sample multiple data observations or rows from the original data, builds decision trees for each bootstrap sample, then integrates predictions of multiple decision trees, and finally uses majority vote or averaging to arrive at final predictions. The RFR) is conceptualized as a strong predictor combining a bunch of weak predictors.

To our knowledge there has only been one paper where machine learning has been applied to predict vaccine utilization and/or demand (Fruggiero et al., 2012). In that study, the researchers used a combination of autoregressive integrated moving average model and neural networks to forecast annual MMR demand in Taipei County, Taiwan. Specifically, the authors use 10 features to build a decision model, using data related to vaccine demand relative variables and population growth relative variables. Our study differs from theirs in several respects— 1) their goal is to forecast annual demand whereas we aim at forecasting bi-weekly demand, 2) they aim at forecasting demand using variables related to population or stock. No features related to health facility location or the facility itself were included. Our model is significantly more comprehensive, as it includes many granular details of the health facilities including their geo-location, altitude, facility details and 3) they aim at forecasting county wide demand whereas we forecast health facility level demand. Our model is therefore, significantly more fine grained.

Vaccine campaigns sometimes deliver vaccines directly or serve to increase demand. Here, we did not have information on vaccine campaign data. In the next round of data gathering, we may be able to access that data and build an increasingly multidimensional, and more accurate model.

## Conclusion

In summary, we present for the first time an interpretable predictive model to forecast vaccine utilization that has a broad scope and can be adapted to many countries and regions. Our study underscores the importance of applying machine learning on hard-to-gather, temporally and spatially relevant integrative datasets to make accurate vaccine utilization forecasts. Importantly, we also present two different versions of a predictive model. RFR model 1 has high predictive performance and can be used in places where recent vaccine utilization data is available. RFR model 2 has slightly less predictive performance but can easily be adapted to other regions and countries. It has broader application scope. This is a tool that can help translate the Global Vaccine Action Plan for 2011–2020 into action: meeting vaccination coverage targets in every region, country and community and strengthening health systems by empowering program managers with access to high quality information on stock needs at each specific location.

## Data Availability

The data analyzed in this study is subject to the following licenses/restrictions: We will share all data referenced here upon permission of the United Republic of Tanzania, anyone who wishes to see the data must first gain permission from the rightful owner of the data, the Government of Tanzania. Requests to access these datasets should be directed to the Government of Tanzania.

## References

[B1] Breiman Friedman (1984). Classification and regression trees. Abingdon‐on‐Thames, Oxfordshire, UK: Taylor and Francis

[B2] Breiman L. (2001). Random forests. Mach. Learn. 45 (1), 5–32. 10.1023/a:1010933404324

[B3] Fruggiero F. Iannone R. Martino G. Miranda S. Riemma S. (2012). “A forecast model for pharmaceutical requirements based on an artificial neural network,” in Proceedings of 2012 IEEE international conference on service operations and logistics, and informatics, Suzhou, China, July 8–10, 2012 (Piscataway, NJ: IEEE), 263–268.

[B4] Pérez F. Granger B. E. (2007). IPython: a system for interactive scientific computing. Comput. Sci. Eng. 9 (3), 21–29. 10.1109/mcse.2007.53

[B5] GPS Visualizer. Available at: http://www.gpsvisualizer.com/elevation (Accessed March 28, 2019).

[B6] Howard J. Gugger S. (2020). fastai: A layered API for Deep Learning. Information 11 (2), 108, Available at: https://github.com/fastai/fastai

[B7] Logistimo; Products. (2011). Available at: https://www.logistimo.com/product.html (Accessed January 16, 2019).

[B8] Lydon P. Schreiber B. Gasca A. Dumolard L. Urfer D. Senouci K. (2017). Vaccine stockouts around the world: are essential vaccines always available when needed? Vaccine 35 (17), 2121–2126. 10.1016/j.vaccine.2016.12.071 28364919

[B9] Mueller L. E. Haidari L. A. Wateska A. R. Phillips R. J. Schmitz M. M. Connor D. L. (2016). The impact of implementing a demand forecasting system into a low-income country's supply chain. Vaccine 34 (32), 3663–3669. 10.1016/j.vaccine.2016.05.027 27219341 PMC4930702

[B10] Orenstein W. A. Ahmed R. (2017). Simply put: vaccination saves lives. Proc. Natl. Acad. Sci. U.S.A. 144 (16), 4031–4033. 10.1073/pnas.1704507114 PMC5402432 28396427

[B11] Ozawa S. Clark S. Portnoy A. Grewal S. Brenzel L. Walker D. G. (2016). Return on investment from childhood immunization in low- and middle-income countries, 2011–20. Health Aff. 35, 199–207. 10.1377/hlthaff.2015.1086 26858370

[B12] Patel P. B. Rana J. J. Jangid S. G. Bavarva N. R. Patel M. J. Bansal R. K. (2015). Vaccine wastage assessment after introduction of open vial policy in surat municipal corporation area of India. Int. J. Health Pol. Manag. 5 (4), 233–236. 10.15171/ijhpm.2015.208 PMC4818988 27239864

[B13] Path and World Health Organization (2011). Developing a vision for immunization supply systems. in 2020: landscape analysis summaries. Available at: http://www.path.org/publications/files/TS_opt_vision_2020.pdf (Accessed January 16, 2019).

[B14] Pedregosa F. Varoquaux G. Gramfort A. Michel V. Thirio B. (2011). Scikit-learn: machine learning in Python, J. Mach. Learn. Res. 12, 2825–2830.

[B15] Python Software Foundation (2018). Python language reference, version 3.6. Available at: http://www.python.org.

[B16] Rajgopal J. Connor D. L. Assi T.-M. Norman B. A. Chen S.-I. Bailey R. R. (2011). The optimal number of routine vaccines to order at health clinics in low or middle income countries. Vaccine 29, 5512–5518. 10.1016/j.vaccine.2011.05.044 21624419 PMC3138835

[B17] Seaborn 0.8.1. Available at http://seaborn.pydata.org/index.html (Accessed January 16, 2019). 10.1007/978-1-4612-0689-7

[B18] Tanzania Health Ministry Registry. Available at: https://hfr-portal.ucchosting.co.tz/ (Accessed March 28, 2019).

[B19] Tanzania National Bureau of Statistics. Available at: https://www.nbs.go.tz/ (Accessed March 28, 2019).

[B20] John Snow, Inc. and USAID (2010). Pipeline 5.1. Available at: http://deliver.jsi.com/John Snow, Inc & Usaid (Accessed January 16, 2019).

