# Supplementary figures and images for: An Interpretable Predictive Model of Vaccine Utilization for Tanzania
Source: Front Artif Intell. 2020 Oct 30;3:559617. doi: 10.3389/frai.2020.559617 (PMC7944351; doi:10.3389/frai.2020.559617)

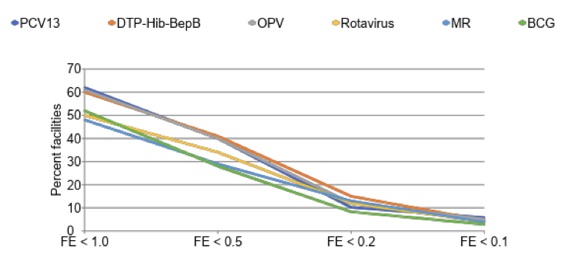

Supplement: Supplementary file 1 [file image1.jpeg]

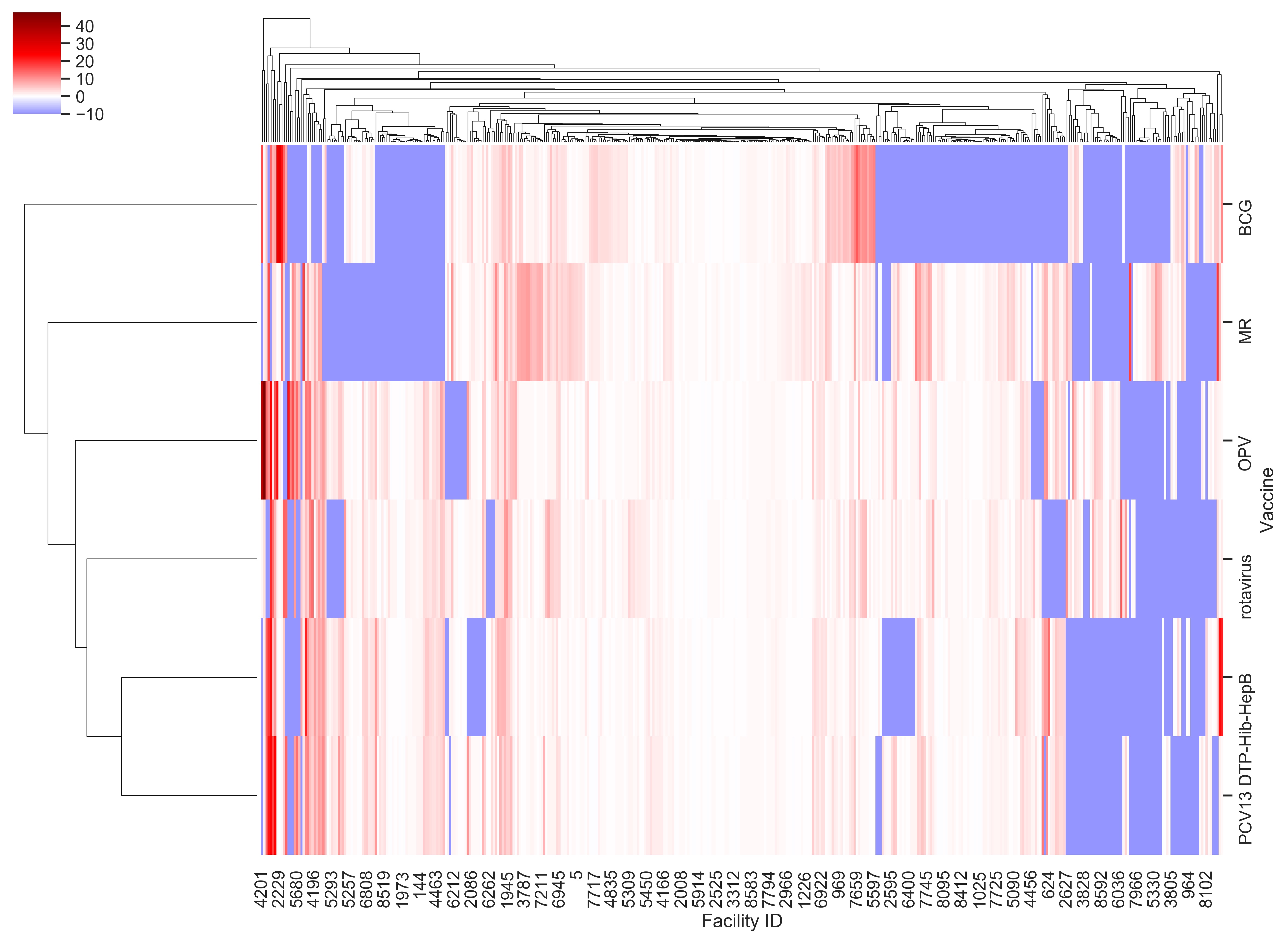

Supplement: Supplementary file 2 [file image2.jpeg]

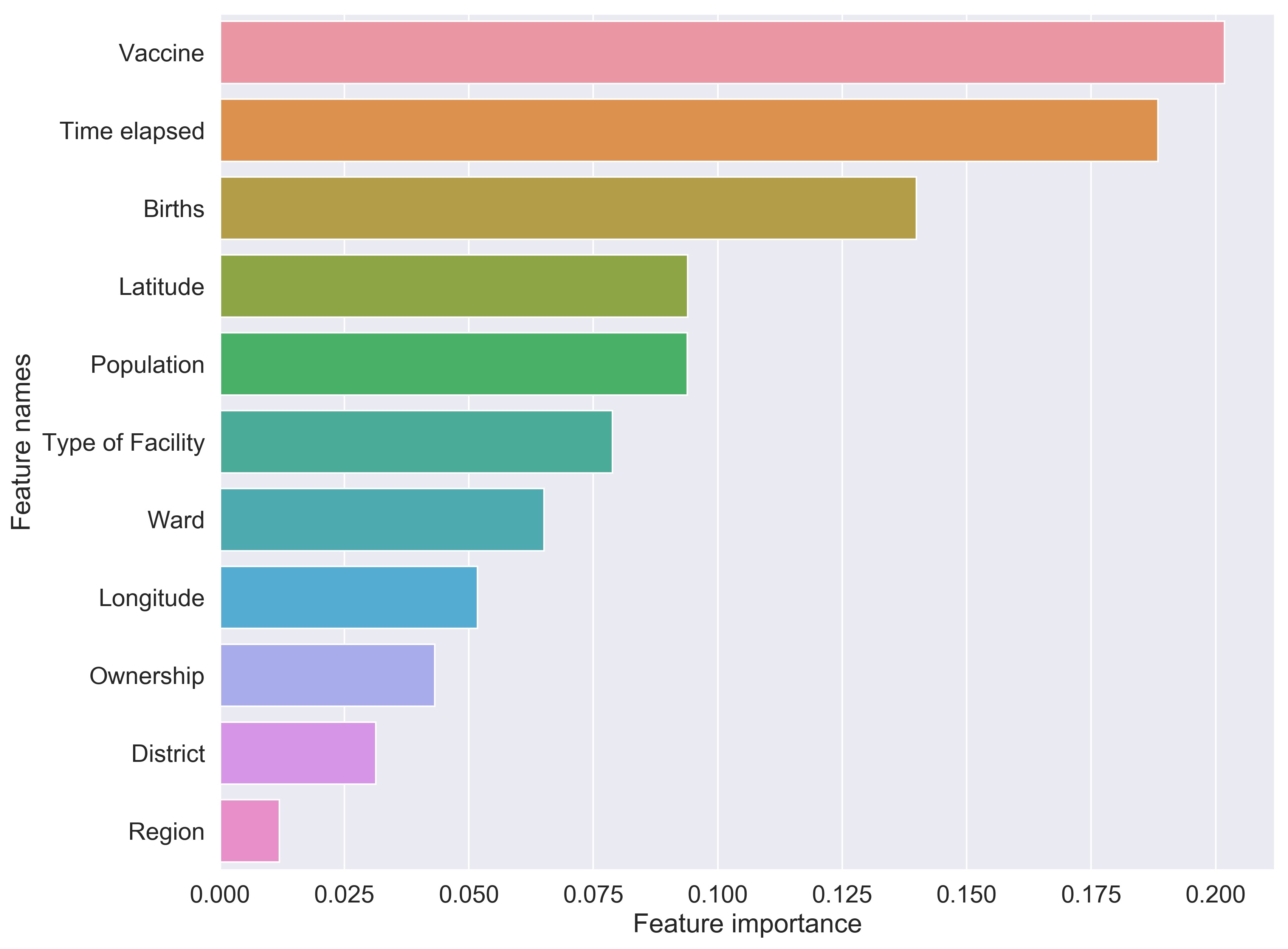

Supplement: Supplementary file 3 [file image3.jpeg]

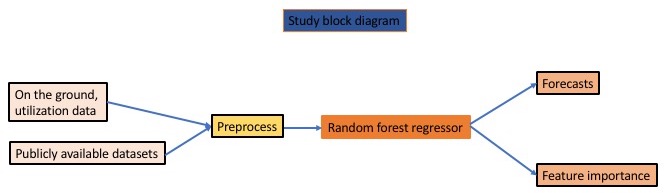

Supplement: Supplementary file 4 [file image4.jpeg]
